# Human immunodeficiency virus awareness and condom use among female adolescent prostitutes in Lagos, Nigeria

**DOI:** 10.11604/pamj.2018.31.178.17034

**Published:** 2018-11-14

**Authors:** Babatunde Ajayi Olofinbiyi, Sogo Angel Olofinbiyi, Oladele Simeon Olatunya, Babatunde Olaniyi Rosiji, Rebecca Oluwafunke Olofinbiyi, Olunike Blessing Olofinbiyi, Olabode Oluwadare Akintoye, Oluwole Dominic Olaogun

**Affiliations:** 1 Department of Obstetrics and Gynaecology, Ekiti State University College of Medicine, Ado-Ekiti, Nigeria; 2 Adolescent Friendly Research Initiative and Care, Nigeria; 3 Criminology and Forensic Studies, School of Applied Science, College of Humanities, University of Kwazulu-Natal, Durban, South Africa; 4 Department of Paediatrics, Ekiti State University College of Medicine, Ado-Ekiti, Nigeria; 5 State Specialist Hospital, Ikole-Ekiti, Nigeria; 6 Department of Nursing, Ekiti State University Teaching Hospital, Ado-Ekiti, Nigeria; 7 School of Public Health and School of Nursing, College of Health Sciences, Howard College Campus, University of KawaZulu-Natal, Durban, South-Africa; 8 Department of Physiology, Ekiti State University College of Medicine, Ado-Ekiti, Nigeria

**Keywords:** Adolescent, prostitutes, HIV, awareness, condom

## Abstract

**Introduction:**

The cornerstone of HIV prevention among female adolescent prostitutes is awareness promotion complemented with advocacy on consistent and correct use of condom. The study aimed at reviewing HIV awareness and condom use among female adolescent prostitutes in Lagos communities, Nigeria.

**Methods:**

It was a mixed-method study realized through a questionnaire-based survey and in-depth interviews of adolescent sex workers in Oyingbo and Yaba communities of Lagos State, Nigeria; conducted between 1^st^ of April, 2014 and 30^th^ of September, 2014. SPSS version 17.0 and content analysis were used in analyzing quantitative and qualitative data respectively.

**Results:**

97.3% had heard about HIV/AIDS; with 86.9% being tested for HIV in the preceding 6 months. While there was consistent use of male condom in 99.7% of the respondents, 90% had experience with the use of female condom, however, 95.5% would allow non- use of condom for higher financial reward.

**Conclusion:**

Although the level of awareness of HIV/AIDS was high among the respondents, there is need to improve on the level of awareness and preventive strategies for HIV/AIDS, with more emphasis laid on the consistent and correct use of condom in this highly vulnerable class of people.

## Introduction

Female adolescent prostitution is a variant of community-induced sexual violence and oppression against young girls and teenagers in the age group of 10-19 years either for sexual gratification or economic reason [1, 2]. It is of both public and reproductive health importance. It is a criminal and antisocial sexual behavior of enormous physical, mental and social burden that cuts across all communities [2, 3]. Human immunodeficiency virus (HIV) infection along with other sexually transmitted infections is one of the most dreaded major medical complications associated with prostitution business [4-6]. Prostitutes and other sexually active young people with risky health behaviour have been shown to be significant HIV transmitters, with HIV prevalence in them almost eight times the national prevalence [4, 5, 7]. Therefore, there is a strong association between this afore-stated finding and the fact that the first case of acquired immune deficiency syndrome (AIDS) in Nigeria was identified in a sexually active 13-year-old girl in 1984 [8]. Health and other social risks associated with the pandemic of HIV scourge include opportunistic infections, organ damage, stigmatization, psychological/mental stress, suicidal ideation and death [8]; all culminating in negative effects on nation’s growth and development. Condom use has been shown to be a reliable HIV infection prevention strategy if used consistently and correctly [9]. It is, however, interesting and pathetic to find out that, despite their high risk sexual behavior, there is high level of inconsistent use of condom among commercial sex workers, thus exposing them much higher to the risk of contracting and spreading HIV infection. It is also equally disturbing that an increasing number of adolescent girls are entering the sex trade; these are regarded as sex slaves who are highly vulnerable to HIV infection because they lack sex negotiation skills especially when dealing with older men [10]. Condom use in sex industry has been influenced by factors such as age of clients, age of sex worker, types of clients (casual or regular), location of business, number of clients, charges, involvement of threat or coercion [11]. Most of the available studies on HIV awareness and condom use in sex industry were conducted generally on men and women of reproductive age (15-49) [11, 12], there is paucity of specific data on the views and experiences of adolescent girls in the same sex trade. This type of study becomes essential to be able to appreciate the possible prostitution peculiarities and conditions in a younger age group (adolescents) that findings have shown that clients have preference for in the business [13]. Information obtained from this study will give a better understanding to the control and prevention of the higher health burden of HIV/STIs in these highly vulnerable people.

## Methods

A research design was drawn to examine the topic of the study and determine the most appropriate methods employed to gather correct and objective data for the study. It was a mixed-method study realized through a questionnaire-based survey and in-depth interviews. The basis of this method was to generate both quantitative and qualitative pieces of information for the study. The questionnaire comprised information reflecting the socio-demographic characteristics of the respondents, level of HIV awareness and condom use among the respondents. The questionnaire was adopted from previous similar studies on prostitution business and it was pretested and validated before administration on the field.

The study population covered those female adolescent sex workers within the age bracket of 10- 19 years in Oyingbo and Yaba communities of Lagos State, Nigeria. It was a 6-month study carried out between 1^st^ of April, 2014 and 30^th^ of September, 2014. Ethnical approval, with protocol reference number 15/00049, was given by the Faculty of Social Sciences, Department of Sociology, University of Lagos, Nigeria. A total number of 300 copies of questionnaire were administered to the female adolescent sex workers in the survey. A total of 300 female adolescent sex workers from 10 different brothels were interviewed in the general survey through a method of personal interview (230 female adolescent sex workers from 6 brothels in Oyingbo community and 70 female adolescent sex workers from 4 brothels in Yaba community). The questionnaire were made anonymous and the women were at liberty to withdraw from the study; all the respondents gave consent. The survey questionnaire was structured in such a way that adequate information was elicited on research questions and objectives, and the questionnaire was arranged in sections to capture the objectives of the study.

However, in the qualitative research method, a total of four (4) in-depth interviews were conducted in the study using unstructured interview with “quick questions”; with the 4 respondents drawn from two sampled local government areas. Each of the respondents (adolescent prostitutes) interviewed were picked from 4 different brothels, two from each of the study communities. This was done to have a more objective and balanced representation of the views of respondents from the two communities of study. This made the data generated from the technique represent a relatively true picture of adolescent prostitution in Lagos metropolis.

A purposive sampling technique was utilized to select respondents in the survey, while in the in-depth interview, respondents were selected using both purposive and accidental sampling techniques (the selected method was based on convenience, chance, availability and relevance of the respondents to the theme of the study). Both descriptive and content analyses were utilized to analyze the data generated. For qualitative analytical method, Statistical Packages for Social Sciences (SPSS/PC+) was used to analyze the collected data. The qualitative data were analyzed using “content analysis”. This technique facilitated compressing inferences, by systematically and objectively identifying specified characteristics of messages. In doing this, the in- depth interviews recorded into the tapes were transcribed from a local language (i.e. Yoruba, Igbo or pidgin) to English language. Responses to each question were summarized and important questions were reported verbatim to complement our findings in the survey. It should be noted that the content analysis was done manually in order to enhance the explanatory clarity of findings.

## Results

Table 1 shows the socio-demographic characteristics of the respondents. While majority of the respondents were between 15 and 19 years, their mean age was 16.9 years. Two hundred and sixty three (90.7%) of the respondents had secondary education, 19(6.6%) primary and 3(1.0%) had no education.

**Table 1:** Socio-demographic characteristic of respondents

	Frequency	Percentage (%)
**Age Group**		
10-14 years	5	1.7
15-19 years	285	98.3
**Religion**		
Christianity	284	97.9
Islam	5	1.7
No Response	1	0.3
**Ethnic Background**		
Yoruba	4	1.4
Igbo	128	44.1
Hausa/Fulani	1	0.3
Others (specify)	157	54.1
**Educational Attainment**		
None	3	1.0
Primary	19	6.6
Secondary	263	90.7
Tertiary	5	1.7
**Current Marital Status**		
Single	283	97.6
Married	4	1.4
Divorced	3	1.0
**Nationality**		
Nigerian	279	96.2
Others (Specify)	11	3.8

Table 2 reveals respondents’ level of HIV awareness. While 252 (86.9%) of the respondents were tested for HIV in less than 6 months to the study period, 16 (5.5%) had the test more than 12 months to the study and 6 (2.1%) showed no response.

**Table 2:** Level of HIV awareness among the respondents

	Frequency	Percentage (%)
**Knowledge about HIV/AIDS**		
Yes	282	97.3
No	1	0.1
No Response	7	2.4
**Source of information on HIV/AIDS**		
Health Workers/Clients	75	25.9
Television/Radio	16	5.5
News Paper/NGO/Friends at Work	7	2.4
Health Sensitization/Health Workers	4	1.4
Health Workers/TV/Radio/Health Sensitization	186	64.1
No Response	2	0.7
**The last time for the test for HIV/AIDS**		
Less than 6months	252	86.9
6-12months	6	2.1
More than 12 months ago	16	5.5
No response	16	5.5

Table 3 shows the level of utilization of condom among the respondents. While 289 (99.7%) of the respondents used male condom with clients, 1 (0.3%) had never used; with 289 (99.7%) using it frequently. While female condom was used by 261 (90%) of the respondents, 29 (10%) had no experience with it; with frequent use recorded in 16 (5.5%) of the respondents. Two hundred and forty-six (84.8%) of the respondents would allow non-use of condom for higher financial reward. While 277 (95.5%) of the respondents believed that education could influence the correct and consistent use of condom among female adolescent sex workers, 11 (3.8%) did not agree that education could be of any value. A larger percentage of the respondent 288 (99.3%) believed that the use of condom could reliably prevent HIV infection.

**Table 3:** Condom use among the respondents

	Frequency	Percentage (%)
**Use of male condom with clients in the business**		
Yes	289	99.7
No	1	0.3
**Frequent use of male condom**		
Always	289	99.7
Not always	1	0.3
**Use of female condom with clients in the business**		
Yes	4	1.4
No	186	64.1
**Frequent use of female condom**		
Always	16	5.5
Not always	250	86.2
No response	24	8.31
**Non-use of condom on higher financial reward**		
Yes	246	84.8
No	41	14.1
No response	3	1.0
**Influence of education on the consistent use of condom among female adolescent sex workers**		
Yes	277	95.5
No	21	3.8
No response	2	0.7
**Way to prevent HIV**		
Use of condom	288	99.3
No response	2	0.7

### Deep in-depth interview on the use of condom

The influence of financial gain on the use of condom

A 14- year respondent said: *“More money is attractive to the economically starved sex workers and condom use can be traded for the extra cash. Clients believing that condoms interfere with sexual pleasure are willing to pay more to avoid condom use. Money, myths and wrong beliefs about safe sexual behavior and protective acts place the sex workers in potential danger by not adopting safer sex norms and not insisting on condom use all the time. When he tempts me with money and drinks, I compromise on the rubber”*.

The influence of education on condom use

A 16-year respondent expressed: *“Education has nothing to do with the use of condoms by adolescent sex workers. As we are all in this business, I can tell you that both literate and illiterate members of this brothel have a better understanding of the use of condom. Even in villages around the country, where people are stark illiterates, yet they consistently use condom for sexual practice. Any prostitutes that want to prove her level of education as regards condom use is only playing with her business, while hunger and suffering may be the reverse of the act. Either you are educated or not, whatever the request of the clients will determine your choice of condom. I know that the use of condom is a universal campaign to ensure safer sex for all”*.

The level of familiarity with clients and condom use

A 15-year-old respondent expressed her views as follows: *“I don’t usually use condom with old customers because I already know them and am used to them and they are used to me. However, I must use condom for a new customer because I am seeing him for the first time”.*

The influence of perceived client’s personal hygiene on the use of condom

A 17-year-old respondent gave the following views: *“I may decide not to use condom if I discover that a client is very dirty because he may have HIV. But if he is clean, I usually waive condom because he is not likely to have disease”*.

## Discussion

The level of awareness of HIV/AIDS was high among the sex workers in the study location; as the findings from this study showed that 282 (97.3%) of the respondents that participated in the research work had heard about HIV/AIDS as a sexually transmitted disease, while 1 (0.3%) had not heard about the disease. This indicates that the risk perception was perfectly high among the adolescent prostitutes; this agreed with Oyefara’s finding that 82.0% of female sex workers had knowledge about the existence of HIV/AIDS [14]; and this shows that the level of HIV awareness among sex workers has increased from 82.0% as of 2007 to 97.3% in the year 2014. Different organizations such as governmental and non-governmental organizations (NGOs); such as World Health Organization (WHO), United Nations International Children Emergency Fund (UNICEF), National Aids Control Agency (NACA) and so on, are working with sex workers in Nigeria and their work has positively impacted on the awareness of the sex workers [15-17]. About 86.9% of the brothel-based sex workers interviewed in the study had been tested for HIV/AIDS in the last six months of their stay in the sex industry. Since the sex workers are scattered in the city of Lagos rather than being concentrated, it will be a great challenge to plan effective screening interventions reaching all their hidden populations.

Although various segments of Nigeria populace/media are doing appreciably well in the area of dissemination of HIV/AIDS information, the health sectors/workers, being the central player, still need to do more as an agent of information dissemination on HIV/AIDS. This becomes necessary as only 4 (1.4%) of the respondents got the information through health sensitization/health workers.

The outcome of the research also revealed that almost all the respondents (99.7%) that participated in the research work used male condom in the business. This is in line with the findings of Masresha *et al.* and Tamene *et al.* where much lower level of condom utilization was reported among female sex workers [18, 19]. This disparity might be a reflection of the difference in sex charges, nature of environment and number of clients. It is also interesting to find out that appreciable segment of the respondents had some experience with the use of female condom, a relatively new invention in barrier contraception; a similar study conducted by Andrews *et al.* only gave a recommendation of including female condom in condom intervention efforts [19].

Although there was high level of consistent use of condom among the sex workers, it is not surprising to find out that most of the respondents 246 (84.8%) would, even upon adequate information and education, still compromise the use of condom in the face of huge financial gain because many people are into prostitution business in order to make ends meet. This is in agreement with the findings of Tamene *et al.* [18].

Most of the sex workers (99.3%) agreed that the use of condom could prevent HIV infection. This is probably reflecting the high level of consistent use of condom among the respondents. A striking part of the strength of this study is that, it is one of the few studies, combining both quantitative and qualitative qualities, on HIV awareness and condom use in sex industry in sub-Saharan Africa; primarily conducted on adolescent prostitutes. Secondly, the relatively large sample size of the study is an added strength; as it is difficult to access and get information from this highly secretive class of people. A major limitation during encounter with the sex workers was their initial reluctance to release information for fear of police arrest, insecurity, invasion of privacy and fear of stigmatization, and that related studies conducted by other researchers did not yield them monetary reward. The problem was solved by establishing more rapport with them for build of confidence and trust; this was complemented with monetary gifts, given not only to the sex workers but also to their madams (their bosses/controllers). In addition, during the course of the in-depth interview, some of the respondents initially frowned at having their voices recorded but with persistent visits, persuasion and reassurance, they were convinced of no harm. Furthermore, problem was encountered in getting some of the respondents’ local languages translated to English language as some of them did not understand English language.

Therefore, more studies of larger sample size are still required to be able to discover more objective findings on HIV infection and condom use in adolescent prostitution, especially in the area of detection of retroviral status, treatment and prevention. Furthermore, it becomes imperative to look into the area of male adolescent prostitutes as this may reveal new and hidden findings in prostitution business.

## Conclusion

Education and empowerment of the girl child with access to the right information on sexually transmitted infections is the cornerstone for reducing the scourge of HIV infection among female adolescent prostitutes. There is need to improve on the level of awareness and preventive strategies for HIV/AIDS especially among adolescent prostitutes. Promotion of correct and consistent use of barrier contraception (majorly condom) will go a long way in preventing and controlling HIV infection in this highly vulnerable class of people.

### What is known about this topic

- HIV awareness is on the increase worldwide;
- Prostitution cuts across races and it is of public health importance;
- Condom use is a reliable tool for HIV prevention.

### What this study adds

- Voluntary HIV testing level is high among this young group of sex workers;
- Financial gain could bring about compromise in condom use among adolescent sex workers despite its invaluable role in HIV prevention.

## Competing interests

The authors declare no competing interests.
